# Transdiagnostic EEG Signatures in ASD and ADHD: A Comparative Review of Computational Biomarkers and Neuromodulatory Interventions

**DOI:** 10.3390/brainsci16090912

**Published:** 2026-08-27

**Authors:** Akshay Bhuvaneswari Ramakrishnan, Nithish Kumar NavaneethaKrishnan, William Mahler, Adrian Schoech, Meenalosini Vimal Cruz

**Affiliations:** 1 School of Computing, Allen E. Paulson College of Engineering and Computing, Georgia Southern University, Statesboro, GA 30460, USA; ab66943@georgiasouthern.edu; 2 Department of Information Technology, Allen E. Paulson College of Engineering and Computing, Georgia Southern University, Statesboro, GA 30460, USA; nn04805@georgiasouthern.edu (N.K.N.); wm07256@georgiasouthern.edu (W.M.); as51735@georgiasouthern.edu (A.S.)

**Keywords:** autism spectrum disorder (ASD), attention-deficit/hyperactivity disorder (ADHD), electroencephalography (EEG), theta/beta ratio (TBR), neurofeedback, mindfulness-based interventions, machine learning, neurodevelopmental disorders

## Abstract

**Highlights:**

**What are the main findings?**

- Theta excess, reduced alpha modulation, and flattened aperiodic slopes recur across ASD and ADHD, but direct comparative studies account for only ~3% of the literature.
- Mindfulness and neurofeedback reliably change EEG, yet symptom improvement attenuates under blinded outcome assessment.

**What are the implications of the main findings?**

- Evidence supports group-level association and diagnostic discrimination; individual-level treatment-response prediction remains unvalidated.
- EEG-guided intervention selection is a testable model requiring prospective randomized validation, not current clinical guidance.

**Abstract:**

**Background/Objectives:** Autism spectrum disorder (ASD) and attention-deficit/hyperactivity disorder (ADHD) are frequently co-occurring neurodevelopmental conditions with partially overlapping neurophysiological profiles. Electroencephalography (EEG) provides non-invasive access to candidate biomarkers, yet the literature remains largely organized around single-diagnosis frameworks, limiting comparison across conditions and constraining translation into intervention selection. This review compares EEG signatures across ASD and ADHD from a transdiagnostic perspective and examines how such signatures might inform the selection of non-pharmacological interventions. **Methods:** A structured search of PubMed, Scopus, IEEE Xplore and Web of Science identified peer-reviewed studies published between 2010 and 2026 reporting EEG findings in ASD and/or ADHD, spanning resting-state, task-based, connectivity, event-related potential, machine learning and intervention studies. Sixty-eight sources were synthesized thematically. Given substantial heterogeneity in acquisition parameters and analytic pipelines, evidence was integrated interpretively rather than pooled quantitatively, and no formal risk-of-bias assessment was undertaken. **Results:** Shared features across both conditions frequently included low-frequency theta excess, reduced alpha modulation under cognitive load, and flattened aperiodic (1/f) slopes—a pattern compatible with, though not a direct measurement of, altered excitation/inhibition balance. While substantial heterogeneity exists, disorder-specific signatures often comprised the ASD “U-shaped” spectral profile alongside elevated epileptiform activity, and frontally pronounced theta/beta ratio elevation in subsets of individuals with ADHD. Machine-learning studies increasingly emphasize interpretable, multidomain feature sets over binary classification. Mindfulness-based and neurofeedback interventions converge on theta reduction and alpha enhancement, although reported effects are frequently conditional on responder status, task context, or outcome-rater blinding. **Conclusions:** Convergent EEG features support a transdiagnostic account of neurodevelopmental dysregulation. A biomarker-informed framework for intervention selection is proposed, which requires prospective validation before clinical application.

## 1. Introduction

Electroencephalography (EEG) has emerged as a valuable non-invasive tool for examining the neurophysiological characteristics of neurodevelopmental disorders, particularly Autism Spectrum Disorder (ASD) and Attention-Deficit/Hyperactivity Disorder (ADHD). In ASD, resting-state EEG studies frequently report atypical neural activity, including altered synchronization, reduced alpha power, and increased delta and theta activity. Some studies describe a “U-shaped” spectral profile, characterized by elevated power at both low- and high-frequency ranges, though this pattern is highly variable and appears most prominent during early development. These neurophysiological patterns have been associated with core clinical features, including social communication difficulties and affective dysregulation. Task-based EEG studies further indicate differences in executive function and emotional processing, even in cases where behavioral performance appears comparable to neurotypical individuals.

In ADHD, resting-state EEG frequently demonstrates elevated theta power, reduced beta activity, and an increased theta/beta ratio (TBR) in subsets of patients, findings that have been linked to cortical hypoarousal and attentional dysregulation. Variations across clinical subtypes have also been reported, with combined-type ADHD often associated with increased frontal theta activity, while inattentive presentations may show reduced beta power. Task-based paradigms reveal reduced alpha suppression and sustained theta activity during cognitive demand. In addition, event-related potentials (ERPs), including the P300 and contingent negative variation, are frequently attenuated, reflecting impairments in attention and inhibitory control.

Despite the surge in EEG research, the existing literature remains largely fragmented. Recent systematic reviews have demonstrated that deep learning approaches, including CNNs and LSTMs, can support ASD identification through high-dimensional EEG feature extraction [1,2], yet they typically focus on binary classification within a single-diagnosis framework. There is a critical scarcity of comparative frameworks that evaluate these neurodevelopmental conditions transdiagnostically to bridge traditionally distinct diagnostic categories.

This fragmentation reflects a broader pattern of diagnostic siloing in neurodevelopmental research. Recent systematic and technical reviews have advanced high-dimensional feature extraction and single-disorder classification considerably [1,3,4], yet comparative frameworks spanning both conditions remain scarce, and the volume of independent research within each disorder has not been matched by synthesis across them. In particular, the intersection of transdiagnostic EEG markers, computational classification, and intervention outcomes remains largely unexamined, leaving computational diagnostic accuracy poorly connected to the mechanistic selection of non-pharmacological therapies. Table 1 summarizes the primary focus of recent reviews in this area alongside the complementary contribution of the present synthesis.

Considered together, these contributions establish the computational and clinical foundations on which the present review builds. Its complementary contribution lies in integrating transdiagnostic EEG markers with intervention frameworks: by examining comorbid ASD + ADHD profiles alongside single-diagnosis presentations, and by relating machine-learning-derived attention states to intervention adjustment, this synthesis extends existing diagnostic precision toward therapeutic application.

Non-pharmacological interventions, including mindfulness-based approaches and neurofeedback, have been explored for their potential to modulate these EEG patterns. However, the clinical applicability of these approaches remains constrained by dataset size, heterogeneity, and limited real-world validation. In ASD, such interventions have been associated with changes in spectral activity and improvements in behavioral outcomes, including anxiety and social functioning. In ADHD, interventions targeting reductions in theta activity and normalization of the theta/beta ratio have shown potential benefits in attentional performance. The integration of EEG with these therapeutic approaches offers opportunities for objective monitoring and the development of individualized intervention strategies. Importantly, these interventions can be conceptualized not only as therapeutic tools but also as mechanisms for directly modulating disorder-relevant neural dynamics identified through EEG.

This review addresses this translational gap by directly comparing the neurophysiological profiles of ASD and ADHD across resting-state, task-based, connectivity, and ERP domains, integrating contemporary machine learning insights to move toward a personalized, biomarker-guided paradigm. Adopting a comparative and transdiagnostic perspective, the review synthesizes evidence on shared EEG abnormalities including theta dysregulation, impaired alpha modulation, and aperiodic slope alterations alongside disorder-specific signatures, examining how these patterns can inform unified intervention strategies. While comparative EEG literature involving ASD and ADHD exists, the specific integration of direct biomarker comparison, machine-learning-based personalization, and structured evidence levels distinguishing group-level from individual-level prediction remains limited. This review synthesizes these three dimensions together, creating a framework for future validation research. Beyond summarizing established findings, the review evaluates how EEG-informed non-pharmacological approaches, particularly neurofeedback and mindfulness-based interventions, can be guided by individual neurophysiological profiles, and identifies future directions for scalable, biomarker-driven neurodevelopmental care.

## 2. Materials and Methods

This review was conducted as a structured literature synthesis examining electroencephalography (EEG) biomarkers and non-pharmacological interventions in autism spectrum disorder (ASD) and attention-deficit/hyperactivity disorder (ADHD). The objective was to integrate findings across neurophysiological, clinical, and computational domains in order to identify shared and disorder-specific patterns relevant to intervention design. It was not conducted as a systematic review or meta-analysis; accordingly, evidence was integrated interpretively rather than through formal quantitative pooling, and no risk-of-bias assessment was undertaken.

A structured search was performed across four electronic databases: PubMed (MEDLINE), Scopus, IEEE Xplore, and Web of Science (Core Collection). The final database queries were executed between May 17 and May 20, 2026. The search strategy was designed to capture intersections of neurodevelopmental disorders, electrophysiological markers, and advanced computational or therapeutic methods. Search strings combined disorder descriptors (e.g., “Autism Spectrum Disorder,” “ADHD”) with methodological and outcome descriptors (e.g., “EEG,” “fNIRS,” “Machine Learning,” “Deep Learning,” “Neurofeedback,” “Mindfulness”).

The initial search retrieved 1360 records (PubMed: 412; IEEE Xplore: 345; Scopus: 318; Web of Science: 285). After automated and manual removal of 435 exact duplicates, 925 unique records underwent title and abstract screening. Screening and data extraction were conducted by a single reviewer; this is acknowledged as a limitation in Section 7. At this phase, 715 records were excluded for being off-topic, outside the target domain, or animal studies. Of the 210 reports sought for retrieval, 12 were inaccessible. The remaining 198 full-text articles were assessed for exact eligibility. During full-text assessment, 132 articles were excluded for the following specific reasons: wrong population (*n* = 38; e.g., adult-exclusive or non-target neurological conditions without an ASD/ADHD cohort); wrong intervention/methodology (*n* = 45; e.g., relied solely on fMRI or behavioral surveys without AI/ML, neurofeedback, or mindfulness); publication type (*n* = 32; e.g., conference abstracts lacking methodological details or editorials); and insufficient data/transparency (*n* = 17; e.g., failed to report model performance metrics or lacked proper cross-validation).

Eligible sources comprised peer-reviewed articles reporting EEG-based findings in ASD and/or ADHD, encompassing resting-state, task-based, spectral, connectivity, and event-related potential (ERP) analyses. Studies of non-pharmacological interventions principally mindfulness-based approaches and neurofeedback were included where neurophysiological or clinical outcomes were reported. Studies applying machine learning to EEG-based classification or Personalization were incorporated to contextualise emerging computational approaches. Sources were excluded where they lacked EEG-based measurement, addressed pharmacological interventions without neurophysiological outcomes, or provided insufficient methodological detail for interpretation.

During the preparation of this manuscript, the authors used QuillBot for language editing, and Claude (Anthropic, San Francisco, CA, USA) for reference formatting and verification. All references were subsequently checked by the authors against primary sources, and all AI-assisted output was reviewed and edited by the authors, who take full responsibility for the content.

The final corpus comprised 68 sources spanning clinical trials, observational studies, systematic reviews, and methodological investigations. To calibrate transdiagnostic inferences appropriately, the evidence base was categorized by study design:

- Direct head-to-head comparisons evaluating both ASD and ADHD cohorts within the same experimental protocol accounted for only ~3% of the literature.
- Comorbid ASD + ADHD cohort were evaluated in only one empirical study.
- Single-diagnosis studies formed the primary neurophysiological foundation, comprising ASD-only cohorts (~30%, n≈21) and ADHD-only cohorts (~18%, n≈12).
- Indirect and computational literature (~48%, n≈32) comprised single-disorder machine learning architectures, intervention trials, and methodological reviews.

Given this distribution, shared neurophysiological features are primarily inferred via indirect parallel synthesis across single-diagnosis studies, with direct comparative and comorbid investigations highlighted where available to anchor transdiagnostic conclusions.

Throughout this review, the following power metrics are referenced: (1) Absolute power: the raw amplitude (μV^2^) of oscillations in a given frequency band; (2) Relative power: the percentage of total power within a specific frequency band relative to all frequencies (0–100 Hz); (3) Adjusted oscillatory power: power in a frequency band after subtracting the contribution of the aperiodic (1/f) component, isolating band-specific oscillatory activity; and (4) Band ratios (e.g., theta/beta ratio): the ratio of absolute or relative power in one frequency band to another. These metrics are variously employed across the 68-source corpus; definitional choices are retained according to original publications.

## 3. Neurophysiological EEG Signatures in ASD and ADHD

When evaluating the electrophysiological markers reviewed below, it is essential to distinguish between direct comparative evidence, comorbid data, and indirect cross-study inferences. Direct head-to-head empirical comparisons between ASD and ADHD remain exceedingly rare in the literature. To date, only two studies in our corpus have directly compared both clinical cohorts under identical recording protocols: Zhao et al. (2025) [10], who evaluated electrophysiological markers during sustained attention in ADHD (n=30), ASD (n=23), and typically developing controls (n=31); and Canigueral et al. (2022) [11], who uniquely evaluated alpha oscillatory dynamics across ASD, ADHD, and a co-enrolled comorbid ASD + ADHD group alongside controls. Consequently, while shared features such as low-frequency theta excess and alpha desynchronization deficits are discussed transdiagnostically, the majority of these convergences represent hypotheses derived from indirect synthesis across separate, diagnosis-specific studies.

- Operationalizing Transdiagnostic Evidence

In operationalizing “transdiagnostic” signatures, it is important to distinguish among types of evidence supporting transdiagnostic claims. As detailed in Section 2, the evidence base comprises direct head-to-head comparisons (ASD and ADHD within identical protocols; ~3%), single-diagnosis studies (ASD-only ~30% and ADHD-only ~18%), and computational and methodological literature (~48%). This distribution means that most transdiagnostic inferences derive from indirect synthesis across separate ASD-only and ADHD-only studies, rather than from direct comparative evidence. Consequently, observed similarities in frequency-band abnormalities between conditions do not by themselves demonstrate shared etiological mechanisms, but rather suggest overlapping neurophysiological phenotypes that may originate from distinct underlying network pathologies. The distinction between direct comparative evidence and indirect inference is maintained throughout Section 3.

- Confounding and Moderating Factors in EEG Interpretation

Age significantly modulates spectral profiles: resting theta dominance is normative in early childhood but abnormal in adolescence, meaning that combining preschool children, adolescents, and adults risks misinterpreting maturational differences as diagnostic signatures [12]. Furthermore, clinical EEG assessment in neurodevelopmental populations requires strict accounting for multiple developmental and clinical moderators. Beyond medication exposure, sleep architecture abnormalities, and epileptiform activity, interpreting broadband EEG features requires rigorously controlling for intellectual disability, biological sex, and frequently co-occurring conditions such as comorbid anxiety. Consequently, the proposed personalized framework cannot be based solely on diagnostic category and broad EEG bands; future computational models must explicitly integrate these major clinical moderators. Clinical EEG assessment in ASD requires attention to sleep architecture abnormalities, including disorganized sleep spindles and variable epileptiform activity [13], which may influence both spectral and connectivity measures. In ADHD, medication exposure—particularly stimulants—can alter theta/beta ratios and spectral power [14,15], and clinical biomarker integration must account for medication timing and status [14].

Methodological and state-dependent factors further complicate interpretation. Recording state (arousal level, posture, task engagement), sleep quality, and time-of-day effects all influence EEG organization. Furthermore, broader electrophysiological research demonstrates that network topology, brain flexibility, and functional connectome fingerprint stability that is, the reproducibility of an individual’s own network configuration across repeated recordings are highly sensitive to transient states, including sleep deprivation and hormonal fluctuations. The distinction between stable individual (trait) EEG profiles and transient, context-dependent (state) fluctuations remains incompletely characterized in the current literature [16]. Consequently, the group-level EEG profiles discussed below should be interpreted as developmental and contextual patterns rather than stable individual biomarkers. Clinical application requires repeated measurement under controlled, standardized conditions to distinguish trait components from state-dependent fluctuations.

### 3.1. Resting-State Spectral Findings

Resting-state EEG in autism spectrum disorder frequently exhibits increased delta and theta power, decreased alpha power, and a characteristic “U-shaped” spectral profile (elevated power at both low- and high-frequency extremes with a relative alpha dip), particularly prominent in early childhood [13,17,18]. However, these patterns show substantial developmental variation and heterogeneity across individuals; not all individuals with ASD exhibit the complete profile, and spectral characteristics evolve significantly across the lifespan. Meta-analyses and large cohort studies suggest that these deviations are present from infancy and may be associated with later symptom severity, especially in social-communication domains [17,19]. Computational approaches, including machine learning, have been explored to classify ASD using resting-state EEG features, although their clinical utility remains under investigation [2].

In typically developing infants (0–2 years), EEG patterns are dominated by delta (0.5–3.5 Hz) waves, shifting into the theta range (4–7 Hz) in early childhood (2–6 years); in contrast, individuals with autism have been reported to exhibit atypical neural synchronization and EEG patterns during these stages [12]. Abnormalities in ASD include slowing of background activity, asymmetry, disorganized sleep architecture (such as asynchrony of sleep spindles), and paradoxical delta activity [13]. Epileptiform findings in ASD vary substantially depending on recording methodology and population characteristics. In a large, clinically referred cohort of 1014 autistic children monitored with repeated sleep EEG at approximately six-month intervals (6-monthly assessments over extended periods), interictal epileptiform discharges (IEDs) were detected in 85.8% (870/1014) [20]. This prevalence figure must be understood in context: it derives from a selected clinical sample rather than a population-based sample, and reflects the markedly greater sensitivity of repeated sleep EEG recordings to detecting IEDs compared to single or waking recordings, which report IED prevalence ranging from 22 to 60.8% [20,21]. Critically, this 85.8% figure describes interictal epileptiform discharges (EEG abnormalities without clinical seizures), not clinical epilepsy (seizure disorder). Population-based studies of autism using single wake or sleep recordings report notably lower IED prevalence (typically 10–30%), indicating that the repeated-sleep-EEG cohort represents a methodologically distinct and potentially clinically enriched sample. The discrepancy between repeated-sleep sensitivity (85.8%) and single-recording sensitivity (22–60.8%) reflects the properties of the recording methodology rather than indicating that most autistic children have clinically significant epileptiform abnormalities requiring urgent intervention. Furthermore, preschoolers with ASD exhibit a significantly lower aperiodic-adjusted alpha center frequency (*p* < 0.05) [22,23], while adolescents have been reported to show excessive delta (1–4 Hz) and theta (4–8 Hz) activity in frontal-central regions. Notably, randomized controlled trials of EEG biofeedback targeting these developmental EEG deviations have reported measurable neurophysiological shifts in background spectral activity (e.g., reductions in delta/theta power) in responder subgroups of ASD children [24]. However, the relationship between these neurophysiological shifts and behavioral or clinical outcomes remains inconsistent across studies, and the mechanisms accounting for individual responder versus non-responder status remain unclear.

In ADHD, the most frequently replicated resting-state finding is increased absolute and relative theta power coupled with decreased beta power, producing a markedly increased theta/beta ratio that is most pronounced frontally. One prominent theoretical interpretation proposes that elevated theta reflects cortical hypoarousal and altered regulation of the default-mode network [14,25]. However, this interpretation is model-dependent; elevated theta could alternatively reflect immaturity of cortical oscillatory development, reduced cortical pruning, or state-dependent factors including vigilance level during recording, medication exposure, or sleep deprivation factors that substantially influence theta power but are inconsistently controlled across studies. The mechanistic significance of elevated theta in ADHD therefore remains interpretively open and requires converging evidence from pharmacological, developmental, and state-controlled studies to establish. However, theta/beta elevation is not universal; approximately 10–15% of medication-naïve ADHD cases do not exhibit marked elevation [14], and substantial heterogeneity exists across ADHD subtypes. Combined-type ADHD shows the highest frontal theta, whereas inattentive-type presentations may show reduced beta rather than theta elevation [25]. Consequently, TBR should not be assumed as an individual-level diagnostic or therapeutic marker without baseline documentation of elevation.

From a clinical screening perspective, the divergence between these resting-state profiles carries direct practical weight. The shared theta excess across both conditions supports transdiagnostic theta-reduction as a broad intervention target, providing a common neurophysiological rationale for protocols applicable to either diagnosis. The ASD-specific ‘U-shaped’ spectral profile is clinically distinguishable from ADHD and has important neurophysiological implications for intervention design. Additionally, interictal epileptiform discharges are more frequently documented in ASD compared to ADHD, which has clinical relevance. However, several important caveats apply to the interpretation of this finding and its implications for clinical practice:

(1) Distinction between EEG abnormalities and clinical epilepsy: Interictal epileptiform discharges on EEG are not synonymous with clinical epilepsy (seizure disorder). Many individuals with IEDs never develop seizures. The clinical significance of IEDs in the absence of seizure history remains debated in neurology.
(2) Evidence basis for screening recommendations: Routine epilepsy screening (EEG assessment) before non-invasive neuromodulatory protocols (mindfulness, neurofeedback) is a clinical recommendation that requires direct empirical support specifically—evidence that these protocols increase seizure risk in individuals with IEDs or that EEG screening prevents adverse outcomes. Such evidence is not currently available in the literature. While clinical vigilance regarding seizure risk in ASD is warranted, the recommendation for routine EEG screening before initiating non-seizure-inducing behavioral interventions should derive from prospective clinical data, not from cross-sectional EEG prevalence findings.
(3) Broadband spectral assessment: Broadband EEG assessment in ASD (encompassing the full frequency spectrum rather than TBR alone) is scientifically justified on neurophysiological grounds, given the documented ‘U-shaped’ profile. This assessment can incidentally detect epileptiform abnormalities, but the primary rationale for broadband assessment is neurophysiological characterization, not epilepsy screening.

In summary, while interictal EEG abnormalities are more prevalent in ASD than ADHD, this observation does not directly establish a requirement for routine epilepsy screening before all behavioral interventions. Clinical practice should be informed by prospective evidence regarding intervention safety and seizure risk, rather than inferred from EEG prevalence studies. The ADHD-specific frontalized TBR elevation, by contrast, offers a more localized and diagnostically reliable target, one whose intervention implications are explored in detail in subsequent sections.

### 3.2. Task-Based EEG Patterns

Task-based paradigms reveal that individuals with ASD often show normal behavioral accuracy but differences in neural recruitment, including reduced alpha event-related desynchronization (indicating impaired top-down modulation) and atypical theta synchronization during social or emotional tasks [26]. Sustained-attention tasks such as the TOVA demonstrate shared P1 reductions with ADHD but additional differences in later inhibitory components, suggesting that attentional impairments in ASD may be more closely related to top-down control than to basic orienting [10].

In contrast, ADHD task-based studies reveal more nuanced patterns than a simple ‘persistent theta elevation’ account. While elevated resting theta is common in ADHD, task-related dynamics show task-phase and task-demand specificity. Studies separating visual working-memory encoding from recall phases have reported lower alpha, beta, and theta power in children with ADHD relative to typically developing controls during encoding, with beta-band differences becoming less pronounced during efficient encoding phases. This task-phase specificity indicates that frequency-band abnormalities in ADHD are not uniform across all cognitive contexts but rather reflect state-dependent and task-demand-dependent modulation failures. Combined-type ADHD shows more pronounced alpha desynchronization deficits [27], while inattentive-type presentations may exhibit greater theta persistence, though these subtype differences also vary with task demands. Virtual-reality and continuous-performance paradigms in adults suggest that task-related EEG abnormalities persist across the lifespan, though effect sizes and patterns vary with task parameters. Consequently, both ASD and ADHD display impaired modulation of low-frequency (theta) and mid-frequency (alpha) rhythms under cognitive demand, but the specific manifestation depends critically on task phase, encoding efficiency, and task demands rather than representing a uniform neural signature.

Further nuances are observed in trait-based analyses. In studies comparing individuals with high (HAT) and low autistic traits (LAT) during executive functioning tasks, the HAT group perceived their performance as significantly worse despite equal behavioral accuracy. This has been interpreted as reflecting a slower decision-making style characteristic of the autism spectrum that may not be fully captured by standard task time constraints [26]. In typically developing children, higher autistic traits (measured by SRS-2) have also been associated with specific atypical neural activity during dynamic facial emotion processing [28].

The mechanistic distinction emerging from task-based findings is particularly instructive for intervention design. Both disorders fail to regulate theta and alpha under cognitive demand, yet the underlying breakdown differs fundamentally: in ASD, the neural system recruits atypically despite producing behaviorally adequate output, suggesting a compensatory inefficiency rather than a performance deficit. In ADHD, the failure is more directly functional persistent default-mode intrusion actively disrupts task engagement in a way that is both measurable and behaviorally consequential. This distinction implies that interventions sharing a theta-suppression goal may need entirely different delivery contexts: ASD protocols may be more effective when embedded in socially or emotionally relevant tasks that engage the specific networks showing atypical recruitment, whereas ADHD protocols are likely better served by sustained-attention paradigms that directly challenge and train resistance to default-mode interference.

### 3.3. Alpha, Beta, and Gamma Dynamics

Alpha power and its modulation represent one of the most studied yet inconsistently replicated bands in ASD. Posterior alpha has often been reported to be reduced at rest and has been cross-sectionally associated with social- deficit severity [29]. Regarding task-induced alpha dynamics, reduced alpha suppression during cognitive engagement has been theoretically interpreted as reflecting impaired functional inhibition or reduced attentional engagement [29]; however, this interpretation remains model-dependent. Reduced alpha suppression could alternatively reflect differences in task strategy, reduced task engagement (not necessarily inhibitory failure), or age-related differences in alpha frequency and development. Prospective studies directly testing the inhibition hypothesis (e.g., via pharmacological enhancement of GABA-mediated inhibition or computational modeling linking alpha to inhibitory physiology) are required to establish mechanistic validity of the inhibition interpretation. Beta and gamma findings are more heterogeneous, with some studies reporting excess high-beta/gamma (linked to sensory hypersensitivity) and others finding reductions during specific cognitive challenges.

Excessive beta activity has been described as a non-epileptiform abnormality in some children with ASD [13]. In preschoolers, alpha power significantly influences both the occurrence and duration of EEG microstates [23]. Overall, ASD individuals have been reported to show reduced relative and absolute alpha power alongside elevated relative beta and absolute gamma power, supporting the “U-shaped” spectral profile observed in some cohorts [17].

In ADHD, alpha dynamics are characterized primarily by reduced event-related desynchronization, particularly over parietal regions, while absolute beta power may be reduced, especially in inattentive presentations. Gamma-band abnormalities are rarely reported and remain under-investigated. The commonly reported pattern across age and subtype is an immature alpha–beta transition under cognitive demand. The shared reduction in alpha modulation under load, combined with divergent beta/gamma profiles, highlights alpha enhancement as a potential transdiagnostic target for both mindfulness-based interventions and neurofeedback protocols.

The alpha modulation deficit shared across both disorders represents the most consistently replicated transdiagnostic finding in the current EEG literature—a finding with direct implications for both mindfulness-based and neurofeedback protocol design. Where the two conditions diverge, however, is in what surrounds that shared deficit. In ASD, the co-occurrence of elevated beta and gamma power has been theoretically interpreted as reflecting pathological high-frequency arousal activity that potentially underlies sensory hypersensitivity and arousal dysregulation. However, this interpretation requires qualification. Elevated gamma in ASD could alternatively reflect: (1) changes in E/I balance that favor higher-frequency oscillations (consistent with the aperiodic slope interpretation above); (2) reduced spectral filtering or altered frequency-dependent dampening in cortical tissue; or (3) increased measurement artifact from muscle activity, electrode noise, or other non-neural sources (particularly problematic in pediatric populations during naturalistic recording). Furthermore, the mechanistic link between gamma power and sensory hypersensitivity remains hypothetical. Prospective validation would require: (a) demonstrating that reducing high-frequency power via targeted interventions improves sensory symptoms (rather than assuming that because gamma is elevated and sensory symptoms are present, gamma causes the symptoms); (b) showing that sensory- enriched or depleted environments produce predicted changes in gamma and symptoms; or (c) computational modeling linking gamma physiology to sensorimotor gain control. In ADHD, the picture is structurally different the primary challenge is not suppression of excess high-frequency activity but rather the promotion of an alpha-to-beta maturation that cortical development has failed to complete. These are not merely different points on the same spectrum; they represent distinct therapeutic objectives that happen to share one common entry point.

### 3.4. Theta/Beta Ratio (TBR)

The theta/beta ratio has been widely studied as a potential biomarker in ADHD. Meta-analytic evidence from observational and clinical studies in research samples supports TBR elevation in approximately 85–90% of medication-naïve cases, though this estimate is substantially influenced by methodological factors including age, recording conditions, and frequency-band definitions [14]. Cross-sectional correlational studies have reported associations between frontal TBR magnitude and symptom severity; however, these group-level associations do not establish individual-level predictive validity. Regarding treatment response, randomized controlled trials of TBR-targeted neurofeedback [30] and mindfulness-based training [16] have reported TBR normalization in responder subgroups; however, as noted in (Section 4.2.3), these neurophysiological changes are not consistently accompanied by symptom improvement under blinded outcome conditions.

In ASD, TBR appears more variable and less diagnostically specific, although emerging evidence suggests moderate elevation in subgroups with prominent attentional or regulatory difficulties. Computational approaches incorporating TBR alongside other spectral and connectivity features have been explored to differentiate ASD, ADHD, and comorbid presentations, although their clinical applicability remains under investigation [11]. Consequently, while TBR is among the most studied resting-state markers in ADHD, its emerging utility in ASD especially in comorbid cases supports its inclusion for further investigation within transdiagnostic frameworks.

The clinical utility of TBR as a biomarker is therefore diagnosis-, individual-, and measurement-dependent. In ADHD subpopulations where frontal TBR elevation is documented at baseline under standardized conditions (controlled arousal state, consistent recording parameters, defined frequency bands, age-appropriate reference scheme), TBR-reduction may theoretically represent an intervention target. However, multiple caveats must be emphasized. First, TBR findings are substantially influenced by age, arousal, recording condition, medication status, reference scheme, frequency- band definition, and task demands; consequently, the ~85–90% elevation rate cited above should not be interpreted as a stable individual marker but rather as group-level prevalence in controlled research settings. Second, approximately 10–15% of medication-naïve ADHD cases in research samples do not exhibit marked TBR elevation, and this proportion likely increases substantially when measurement parameters vary in real-world clinical settings. Third, and most critically, individual responder status to TBR-targeted interventions remains poorly predicted by baseline TBR metrics alone [15]; baseline EEG does not reliably distinguish who will respond to TBR-reduction protocols versus sham or alternative interventions. Consequently, while baseline EEG assessment may inform intervention selection hypothetically, TBR is not sufficiently robust to guide individual treatment decisions without prospective predictive validation demonstrating that EEG-based assignment produces superior outcomes compared with clinically guided or sham-controlled assignment.

The robustness of TBR as an individual biomarker is further constrained by substantial measurement and contextual confounds. TBR elevation in ADHD is influenced by: (1) age (with greater elevation in younger children); (2) arousal state during recording (task vs. resting, eyes open vs. closed); (3) recording condition (laboratory vs. ecological); (4) reference scheme (linked mastoid vs. average reference vs. source localization), which can substantially alter apparent TBR; (5) frequency-band definitions, which vary across studies (theta typically 4–8 Hz but sometimes 3–7 Hz or 4–7 Hz; beta typically 12–30 Hz but sometimes 13–30 Hz), affecting TBR estimates; (6) task demands and task phase, as shown above; and (7) clinical heterogeneity across ADHD subtypes. This heterogeneity means that whether an individual “shows TBR elevation” depends substantially on how and when the recording was obtained, not solely on their underlying neurophysiology. Clinical implementation of TBR-based intervention assignment would therefore require: (a) standardized recording protocols; (b) age-appropriate normative reference values; (c) repeated measurement under controlled conditions to establish trait versus state components; and (d) prospective validation that EEG-based thresholds predict treatment response. None of these prerequisites have been systematically established in the ADHD literature.

In ASD, TBR appears more variable and less diagnostically specific than in ADHD, with elevation most reliably observed in subgroups where attentional or executive difficulties dominate the clinical picture—presentations that phenotypically overlap with ADHD. TBR monitoring in ASD may theoretically be most relevant precisely in comorbid or ADHD-like presentations, where it could serve as a shared biomarker target between diagnostic categories. However, the same caveats regarding measurement confounds and lack of treatment-response prediction apply equally to ASD. TBR should not be applied as a clinical biomarker for individual treatment assignment in either ASD or ADHD without: (1) standardized recording protocols and frequency-band definitions; (2) test–retest reliability data establishing stability within individuals; and (3) prospective validation demonstrating that baseline TBR predicts individual treatment response.

### 3.5. Functional Connectivity and Coherence

ASD has been reported to exhibit a developmentally shifting pattern: early local hyper-connectivity and long-range hypo-connectivity that may change in adolescence and adulthood [10,31]. Reduced alpha coherence between temporal-parietal and frontal regions has been frequently reported, alongside disrupted default-mode and mirror-neuron network synchrony [32]. In young boys (3–5 years) with ASD, wavelet phase coherence has been shown to reveal significantly reduced functional connectivity in all frontal probe pairs within the theta-alpha band (3.5–12 Hz). Follow-up studies on older boys (5–15 years) suggest this reduced connectivity but localize it to posterior regions [31]. Systematic reviews of EEG/MEG data further highlight abnormal lateralization, specifically elevated left-over-right connectivity ratios [33].

In contrast, ADHD resting-state connectivity studies frequently report increased default-mode network intrusion into task-positive networks, reflected in reduced fronto-parietal coherence and excessive theta-driven long-range coupling. Alpha-band coherence deficits are also prominent and have been associated with attentional lability. Ultimately, both disorders show evidence of dysfunctional long-range integration, albeit in opposite directions at different developmental stages, highlighting the potential relevance of connectivity-informed approaches in future intervention strategies.

Disrupted long-range integration thus emerges as a shared neurophysiological substrate across both conditions, yet the temporal and spatial architecture of that disruption reflects fundamentally different developmental trajectories. In ASD, the connectivity landscape is not static it shifts from early local hyper-connectivity toward long-range hypo-connectivity as development progresses, accompanied by atypical lateralization patterns that suggest a broader failure of network specialization over time. This developmental instability makes ASD connectivity profiles particularly difficult to target with fixed protocols and argues for longitudinal EEG monitoring rather than single-timepoint assessment. In ADHD, functional connectivity studies have documented patterns consistent with greater default-mode network (DMN) activity during task performance compared to typically developing controls. One prominent theoretical interpretation proposes that this represents a pathological ‘intrusion’ of the DMN into task-positive circuits—a failure of task-positive/DMN anticorrelation that disrupts focused attention. However, alternative interpretations warrant consideration. Enhanced DMN activity during tasks could reflect: (1) individual differences in task engagement strategy rather than pathological network interference; (2) developmental immaturity of DMN/task-positive segregation (a normative feature of childhood brain development); (3) increased mind-wandering or task-unrelated thought as a state-dependent phenomenon rather than a trait mechanism; or (4) differences in how the DMN is defined and delineated across studies (different ICA approaches, seed-based definitions, or thresholding procedures yield different conclusions about DMN involvement). Furthermore, the mechanistic link between DMN intrusion and ADHD symptoms remains indirect. Prospective testing would require demonstrating that reducing DMN activity specifically (not just increasing task-positive activity) produces symptom improvement, and that this improvement is mechanistically mediated by enhanced task-positive/DMN segregation rather than by non-specific training effects. Together, these profiles suggest that connectivity-informed interventions are warranted in both disorders, but must be calibrated to fundamentally different network pathologies.

### 3.6. Event-Related Potentials (ERPs)

Classic attentional components, including the P1, N1, and P300, have been reported to be attenuated in both ASD and ADHD. While both disorders share early visual processing deficits evidenced by attenuated P1 amplitudes during vigilance they diverge in later stages: ASD has been associated with differences in early sensory gating, whereas ADHD has been associated with impairments in later allocation and updating. Inhibitory components, such as the N2 and error-related negativity, have also been reported to be reduced across both conditions [26,27]. However, the underlying mechanisms appear distinct, potentially reflecting social-emotional processing deficits in ASD versus motoric inhibition challenges in ADHD.

In reinforcement learning and oddball tasks, no significant differences were reported in the amplitudes of the reward positivity (RewP) or P300 ERP components between high and low autistic trait groups. Reward-processing ERPs, including reward positivity (RewP) and feedback-related negativity, have been associated with blunted responses in both conditions, potentially explaining overlapping motivational difficulties. Interestingly, while some studies found no significant RewP or P300 differences between high and low autistic trait groups during specific reinforcement learning tasks, broader lifespan meta-analyses suggest that P300 and contingent negative variation reductions persist from childhood through adulthood in ADHD. These reductions have also been reported, albeit less consistently, in ASD [26]. Collectively, these shared ERP deficits highlight attention and inhibition as potential shared domains of interest for future intervention-focused research.

The ERP literature ultimately reinforces a pattern that recurs across multiple levels of analysis in this review: a shared early-stage vulnerability giving way to disorder-specific divergence at higher cognitive processing levels. The convergence on attenuated early visual components and blunted reward-related ERPs across both conditions points to a common deficit in basic sensory vigilance and motivational engagement—one that transdiagnostic intervention frameworks can legitimately target without requiring diagnostic differentiation. Where the conditions part ways is in the robustness and longevity of later-stage deficits. The P300 and contingent negative variation reductions in ADHD are among the most consistently replicated ERP findings in the neurodevelopmental literature, persisting from childhood through adulthood and anchoring a clear lifelong profile of impaired inhibitory control and resource updating. In ASD, the equivalent later-stage deficits are present but considerably more variable, shaped more by sensory gating idiosyncrasies and social-emotional processing demands than by a uniform inhibitory failure. For intervention design, this distinction carries a specific implication: while early attentional orienting represents a shared and accessible target, sustaining inhibitory control across the lifespan warrants dedicated protocol attention in ADHD in a way that cannot yet be uniformly justified for ASD.

### 3.7. Spectral Power Abnormalities

High-frequency abnormalities in ASD remain less consistent than low-frequency findings: some studies report variable gamma excess potentially linked to sensory hypersensitivity, while others find no significant elevation. In ADHD, gamma-band abnormalities are rarely reported and remain substantially under-investigated relative to the theta and alpha literature.

The persistence of TBR normalization beyond the training period has been reported in some cases [34]; however, this observation derives from unblinded outcome studies and may reflect expectancy effects, engagement, or regression to baseline rather than persistent neuroplastic change. Critically, whether TBR normalization is causally linked to symptom improvement or represents an epiphenomenon accompanying broader changes in self-regulation or motivation remains unestablished. However, several critical caveats must be emphasized. First, aperiodic slope represents an indirect proxy rather than a direct or uniquely interpretable measure of E/I imbalance. Alternative mechanisms could produce similar aperiodic slope changes, including shifts in synaptic time constants, filtering properties of neural tissue, or changes in the relative contribution of different cell populations. Second, aperiodic slope is substantially influenced by confounds including age (developmental changes in spectral properties), vigilance level during recording (drowsiness flattens spectra), preprocessing choices (filter type and cutoff frequencies alter slope estimates), spectral parameterization method (different algorithms yield different slope estimates), and contamination by residual oscillatory activity. These confounds are inconsistently reported and controlled across studies. Mechanistic validation of the E/I balance interpretation would require direct demonstration through pharmacological perturbation (e.g., GABA agonists or NMDA antagonists affecting aperiodic slope in predicted directions), computational modeling, or within-subject state manipulations showing that E/I perturbations reliably alter aperiodic slope in predicted ways. Unlike traditional band-specific analyses, aperiodic slope may more broadly reflect underlying cortical state dynamics and could inform neuromodulatory intervention design; however, clinical translation requires validation of the mechanistic link between aperiodic slope alterations and specific intervention targets.

Taken together, these findings are summarized in Table 2 and provide the spectral foundation for the transdiagnostic intervention framework described in subsequent sections.

## 4. EEG-Informed Non-Pharmacological Interventions

### 4.1. Mindfulness-Based Interventions

#### 4.1.1. Conceptual Basis of Mindfulness and Neural Self-Regulation

Mindfulness-based interventions encompass a broad range of practices aimed at cultivating present-moment awareness, with QEEG monitoring increasingly used to track their neurophysiological effects in neurodevelopmental populations [35]. These include focused attention meditation, open-monitoring styles like Vipassana, transcendental meditation, mindfulness built into performance tasks [36], standard mindfulness-based stress reduction (MBSR), short standalone exercises [37], and newer tech-supported versions such as VR-based delivery [38]. The theoretical objectives across these approaches are to improve attention control, ease emotional reactivity, and adjust arousal levels—domains that are characteristically dysregulated in ASD and ADHD. However, whether different mindfulness styles achieve these objectives through the proposed mechanisms or through non-specific factors such as engagement, expectancy, or therapeutic relationship remains unclear, and is not yet well-studied in neurodevelopmental populations.

Studies suggest that MBIs can modulate brain activity in neurodivergent populations, producing measurable shifts in power and coherence across specific EEG frequency bands. These practices are generally categorized by their distinct neurophysiological signatures: focused attention and compassion-based styles have been associated with increases in gamma and high-beta activity; open-monitoring practices have been linked to frontal midline theta; and automatic self-transcending styles promote frontal alpha waves [16]. Regular engagement in these practices has been suggested to influence neural coherence and spectral dynamics, often aligning with observable behavioral and emotional improvements.

Ultimately, the conceptual utility of MBIs in neurodevelopmental care lies in their ability to act as a “regulatory mechanism,” where specific meditative styles can be mapped to the frequency-based deficits of the individual. This provides a theoretical bridge for transdiagnostic care, where a single intervention framework can be tuned to address either the low-frequency dominance seen in ADHD or the high-frequency/alpha-dysregulation characteristic of ASD.

#### 4.1.2. Mindfulness in ASD: EEG and Clinical Outcomes

Most studies of adult Mindfulness-Based Stress Reduction (MBSR) in ASD are observational rather than controlled; in one such observational study, MBSR was associated with distinct reductions in frontal and parietal gamma power alongside decreases in frontal beta-2 power [37]. These observed gamma reductions were associated with reductions in clinical anxiety, though the observational design precludes causal inference and attribution of effects to mindfulness specifically versus expectancy or general engagement. Short-term effects have also been documented in feasibility investigations: a brief 2 min mindfulness meditation was reported in an uncontrolled pre-post study to produce measurable changes in resting-state EEG in youth with ASD, including increases in alpha and theta power and decreases in beta power [39]. While these acute changes are neurophysiologically plausible, the lack of control condition limits inference regarding specificity or clinical meaningfulness. Across the available evidence, most studies of mindfulness in ASD are observational or uncontrolled, limiting causal inference. In the few controlled trials and observational studies identified, mindfulness was associated with EEG shifts including increases in alpha power and decreases in high-frequency activity in some individuals [37,39]. These EEG changes could theoretically support the hypothesis that mindfulness acts to enhance alpha-band regularization while reducing high-frequency noise; however, several limitations constrain this interpretation. First, alpha increases and high-frequency reductions have also been documented following unrelated psychological interventions (e.g., play therapy; Section 4.1.2), suggesting that these EEG changes are not specific to mindfulness. Second, in the broader intervention literature, behavioral improvement has not consistently correlated with the magnitude of EEG change a dissociation documented in the play-therapy trial discussed in Section 4.1.2 [40] and comparable mediation analyses have not been conducted for mindfulness specifically. Third, mechanistic validation would require prospective demonstration that increasing alpha or reducing high- frequency activity via targeted neurofeedback independently produces the same behavioral benefits as mindfulness a comparison not yet conducted. Therefore, while mindfulness-associated EEG shifts are neurophysiologically plausible and may be conceptually coherent with the U-shaped profile, the evidence does not yet establish that these EEG changes are mechanisms of behavioral improvement rather than epiphenomena or consequences of non-specific factors like engagement or expectancy. The association between these shifts and reported gains in social- emotional functioning suggests that mindfulness warrants evaluation as a primary rather than purely adjunctive intervention in ASD presentations where sensory arousal dysregulation dominates the clinical picture a proposition that requires testing in adequately powered randomized controlled trials with active control conditions.

- Broader Psychological and Play-Based Interventions in ASD

Beyond mindfulness-based approaches, other psychological and behavioral interventions have been explored for their effects on EEG patterns and clinical outcomes in ASD. In a randomised controlled trial of 65 autistic children allocated to child-Centered play therapy (CCPT; *n* = 34) or a waitlist control condition (*n* = 31), increases in EEG alpha power following intervention were associated with concurrent Behavioral improvement within the intervention arm, with the strength of association varying by recording context. During story- listening, alpha change correlated with reduced social impairment (SRS-2 total: r = −0.72 to −0.88 across parent, stranger and alone conditions, all *p* < 0.001) and with improved adaptive social skills (ABAS-II social domain: r = 0.44 to 0.52, all *p* < 0.01). Corresponding correlations during sand-playing were weaker for SRS-2 (r = −0.52 to −0.60) and did not reach significance for ABAS-II [40].

An important methodological note is that Child-Centered play therapy is fundamentally distinct from mindfulness-based interventions. While both CCPT and mindfulness- based approaches produced measurable alpha modulation in these trials, the underlying mechanisms differ substantially. CCPT operates through child-directed play, therapeutic relationship quality, and behavioral engagement within a supportive context, whereas mindfulness emphasizes metacognitive awareness, present-moment attention, and explicit self-regulatory training. EEG alpha changes following CCPT should therefore not be interpreted as direct evidence for mindfulness-specific neurophysiological mechanisms, but rather as evidence that broader psychological and behavioral engagement can modulate neural activity in autistic children. This distinction has important implications for mechanism-specific hypothesis testing: CCPT and mindfulness may both produce alpha enhancement, but through distinct causal pathways. Claims regarding mindfulness-specific mechanisms require evidence from mindfulness-specific interventions, not from play-based or other psychological approaches that may produce similar EEG outcomes through different processes.

#### 4.1.3. Mindfulness in ADHD: EEG and Clinical Outcomes

VR-based mindfulness has been piloted in a feasibility study in adults with ADHD (*n* = small); preliminary pre-post data suggested improvements in attentional and clinical symptoms, though the lack of control group limits inference regarding specificity [38]. In separate observational studies and open-label trials, traditional MBSR programs were associated with EEG shifts including reductions in theta and increases in beta activity; in some cases, these neurophysiological changes showed partial persistence at follow-up [16]. However, observational designs and lack of blinded outcome assessment limit causal interpretation. Based on the mechanistic targets of mindfulness (theta/alpha modulation) and neurofeedback (theta/beta ratio normalization) reviewed separately in Section 4.1 and Section 4.2, these modalities theoretically engage overlapping frequency domains; however, direct comparative studies within the same sample remain extremely limited [41], and claims of mechanism overlap remain largely speculative pending head-to-head trials.

Structured eight-week mindfulness programmes for adolescents and adults with ADHD have been associated with improvements in attentional performance and reductions in inattention and hyperactivity symptoms [42]. Theta reduction may play a role in addressing the slow-wave dominance typical in ADHD, potentially supporting vigilance and impulse control [43]. Across studies, ADHD-related mindfulness has been reported to target theta/TBR decreases and beta increases in contrast to ASD findings, which more often emphasize higher frequencies or alpha modulation.

Across the available evidence on mindfulness in ADHD, most studies are observational or involve unblinded outcome assessment. In the trials and observational studies available, mindfulness was associated with EEG shifts including theta reductions and alpha/beta increases in some participants [16,42]. One prominent hypothesis proposes that mindfulness operates by reducing frontal theta dominance and enhancing alpha-to-beta regulatory balance—a direction of change that partially parallels changes associated with stimulant medication [16]. However, this mechanistic interpretation requires qualification. First, similar theta reductions and alpha enhancements have been documented following diverse psychological interventions (meditation, cognitive therapy, behavioral training), suggesting these EEG changes are not specific to mindfulness [43]. Second, behavioral improvement following mindfulness was not consistently correlated with the magnitude of EEG change [42]. Third, mechanistic validation would require prospective demonstration that achieving theta reduction via targeted neurofeedback independently produces the same behavioral benefits as mindfulness training—a direct comparison not yet conducted. Therefore, while mindfulness-associated EEG shifts may theoretically target arousal dysregulation relevant to ADHD, the evidence does not yet establish that observed behavioral improvements are mediated by these specific EEG changes versus reflecting non-specific effects of training, engagement, or expectancy. Mindfulness may represent a useful therapeutic option for individuals in whom stimulant medication is contraindicated, but should be understood as potentially beneficial through multiple possible mechanisms, not specifically through theta reduction.

#### 4.1.4. Comparative Impact of Mindfulness in ASD vs. ADHD

While both disorders benefit from the self-regulatory nature of mindfulness, the neurophysiological targets are markedly distinct. In ADHD, the primary objective is “Reduction” specifically the lowering of excessive theta power and TBR to mitigate cortical hypoarousal. Conversely, in ASD, the objective is “Modulation and Suppression,” focusing on the enhancement of alpha-band inhibition and the reduction in high-frequency high-beta/gamma noise linked to sensory hypersensitivity. These divergent pathways suggest that ADHD-focused mindfulness should prioritize vigilance-enhancing, focused-attention styles, while ASD-focused mindfulness may be more effective when utilizing open-monitoring or automatic self-transcending styles that promote global alpha synchronization and emotional calm.

### 4.2. Neurofeedback Interventions

#### 4.2.1. Neurofeedback as a Targeted EEG Modulation Approach

In contrast to mindfulness-based practices, neurofeedback represents a technology-assisted form of brain training in which participants receive real-time EEG feedback to learn to actively modulate specific neural rhythms. This approach can be considered biomarker-guided, as it directly trains the individual to alter measurable patterns of neural activity associated with attentional, emotional, or arousal dysregulation. While the two modalities differ in their primary mechanisms, both aim to improve self-regulation through the modification of dysfunctional EEG dynamics, making neurofeedback a relevant approach for disorders characterized by identifiable electrophysiological abnormalities.

The mechanistic contrast between these two modalities has direct bearing on patient selection. Where mindfulness demands the explicit, top-down cultivation of metacognitive awareness, a cognitive load that may itself be prohibitive for individuals with significant executive deficit neurofeedback, bypasses this requirement entirely, achieving self-regulatory learning through bottom-up operant conditioning via real-time biomarker reinforcement. This is not a trivial distinction: it means that the two interventions are not simply interchangeable options of equivalent accessibility, but rather complementary tools whose relative utility is determined by the individual’s baseline cognitive and regulatory capacity. For clinicians, this reframes the choice between mindfulness and neurofeedback less as a preference question and more as a patient-matching decision grounded in neurophysiological profile.

#### 4.2.2. Neurofeedback in ASD

In a placebo-controlled mu-rhythm training study comprising two experiments (Study 1: *n* = 8; Study 2: *n* = 19), 30 sessions of neurofeedback delivered over approximately ten weeks produced a significant group × category interaction on the Autism Treatment Evaluation Checklist (ATEC) in both experiments (F(3,12) = 4.76 and F(4,68) = 4.82, both *p* < 0.05), although the direction of change across ATEC dimensions differed between studies and individual responses varied considerably, with some participants showing deterioration [44]. Notably, ATEC change was not correlated with mu suppression indices, indicating that Behavioral gains could not be attributed directly to the targeted neurophysiological mechanism.

In a randomised controlled trial comparing EEG- with skin-conductance-biofeedback across 40 sessions, seven of thirteen participants in the EEG-biofeedback arm demonstrated systematic reduction in delta and/or theta power across sessions and were classified post hoc as EEG-regulators. Within this subgroup, improvement in cognitive flexibility was observed on the Trail Making Test (part C minus part B; *p* = 0.017), although the small subgroup size and post hoc responder classification limit the inferences that can be drawn [24].

The mechanisms proposed for neurofeedback in ASD involve targeting low-frequency dysregulation to enhance cortical inhibition, though this mechanistic interpretation remains speculative. Training targeting mu rhythms (alpha band) over 10 weeks has been reported to produce reductions in mu power and increases in attention measures in some responder subgroups [44]; however, the relationship between these neurophysiological changes and behavioral outcomes varies substantially across participants and task contexts. Notably, behavioral gains were not correlated with the magnitude of mu suppression [44], raising questions about whether EEG changes directly mediate behavioral improvement. Sensorimotor Rhythm (SMR) and Slow Cortical Potential (SCP) protocols have been reported to reduce delta power while enhancing alpha activity in some cases [45]. Observational EEG studies in autistic individuals document cross-sectional associations between elevated alpha power and more positive affect, and between reduced delta power and lower levels of negative affect [29]; however, these group-level associations do not establish that training individuals to increase alpha or reduce delta will produce corresponding improvements in affect or behavior. Individual responder heterogeneity in neurofeedback outcomes (with some participants showing behavioral deterioration [44]) suggests that marker modification does not automatically translate to clinical benefit. The theoretical rationale for neurofeedback in ASD rests on the observation that broadband spectral dysregulation (reflected in the ‘U-shaped’ profile) and fragmented long-distance connectivity are frequently observed in the condition. The biomarker-guided reinforcement mechanism allows training to be anchored to individually verified EEG abnormalities rather than standardized protocols. However, a critical distinction must be drawn: identifying a diagnostic feature does not establish causality in symptom generation, nor does modification of that feature guarantee clinical benefit. The U-shaped profile is associated with ASD, but whether correcting the U-shaped profile produces improvements in social-communication deficits remains undemonstrated. Current evidence shows neurofeedback-induced spectral changes in responder subgroups, but does not establish that these changes are causally linked to behavioral outcomes or that non-responders would have benefited from alternative targets.

#### 4.2.3. Neurofeedback in ADHD

Neurofeedback protocols in ADHD primarily target theta suppression, beta enhancement, and the normalization of the frontal theta/beta ratio (TBR) [30]. In individual randomized controlled trials, subsets of participants (responders) show reductions in TBR and increases in attention measures following neurofeedback training [34]; however, responder rates vary substantially across studies, with estimates ranging from approximately 40–70%. A critical caveat must be emphasized: meta-analytic evidence from randomized controlled trials demonstrates that improvements in ADHD symptoms are substantially attenuated, and no longer statistically significant, when analyses are restricted to probably blinded outcome measures rather than unblinded parent or clinician ratings [15]. This dissociation between unblinded and blinded outcomes raises fundamental questions about whether observed TBR changes reflect specific neurophysiological action or accompany non-specific effects such as expectancy, attention, or engagement. Furthermore, baseline TBR elevation does not reliably predict individual neurofeedback response [46], suggesting that TBR status is neither necessary nor sufficient for treatment benefit.

The persistence of TBR normalization beyond the training period has been reported in some cases [34]; however, this observation derives from unblinded outcome studies and may reflect expectancy effects, engagement, or regression to baseline rather than persistent neuroplastic change whether TBR normalization is causally linked to symptom improvement or whether it is an epiphenomenon accompanying broader changes in self-regulation or motivation.

These effect estimates are strongly conditioned on outcome assessment method. A meta-analysis of randomised controlled trials found that improvements in ADHD symptoms were substantially attenuated, and no longer statistically significant, when analyses were restricted to probably blinded outcome measures rather than unblinded parent or clinician ratings [15]. This dissociation represents the central unresolved question in the neurofeedback literature and cautions against interpreting reported effect sizes as direct evidence of specific neurophysiological action. Protocol heterogeneity may itself contribute: trials applying standardised theta/beta protocols to unselected ADHD samples necessarily include participants whose baseline EEG does not exhibit the abnormality the protocol targets. Consistent with this, baseline cognitive and neurophysiological markers have been shown to predict differential neurofeedback response [46]. Biomarker-guided selection therefore offers one testable explanation for the variance observed across trials, and constitutes a specific empirical prediction of the framework proposed here.

Hybrid approaches combining neurofeedback with meditative elements have been proposed as a means of broadening the scope of arousal regulation beyond single-band training [47]. The emergence of low-channel consumer-grade EEG systems has additionally been suggested as a pathway toward improved accessibility, although validation of such devices for TBR-based training remains limited [48].

The clinical interpretation of neurofeedback effects in ADHD remains contested. While some studies report that TBR normalization persists beyond the active training period in responder subgroups, several critical limitations constrain interpretation. Importantly, demonstrating that neurofeedback produces EEG target engagement (TBR reduction) does not establish clinical efficacy; the critical distinction is that EEG changes occur reliably, but behavioral symptom improvement is inconsistently linked to these EEG shifts and disappears under blinded outcome assessment. First, persistence of EEG changes does not establish durability of clinical benefit—the correlation between TBR normalization and behavioral improvement is inconsistent even within responder subgroups [44]. Second, persistence of EEG changes, even if confirmed, does not necessarily indicate neuroplastic adaptation; alternative explanations include expectancy effects, changes in motivation or engagement that persist beyond training, or regression to individual baseline patterns. Third, and most critically, meta-analytic evidence demonstrates that reported clinical benefits of neurofeedback become attenuated or nonsignificant under probably blinded outcome assessment [15], raising fundamental questions about whether observed behavioral gains represent specific treatment effects or reflect non-specific factors including expectancy, demand effects, or clinician allegiance. Whether neurofeedback induces durable neuroplastic change versus producing transient state-dependent benefits remains unresolved pending prospective, blinded, long-term follow-up studies. The emerging integration of neurofeedback with mindfulness-based elements further extends this advantage, combining the data-driven precision of biomarker-targeted TBR reduction with the broader arousal-regulatory benefits of meditative practice an approach whose clinical logic is grounded directly in the complementary mechanisms outlined in Section 4.1.

### 4.3. Transdiagnostic Synthesis and EEG-Guided Intervention Selection

While mindfulness and neurofeedback are predominantly evaluated in separate, diagnosis-specific clinical literature, parallel findings indicate that both modalities target overlapping neurophysiological bandwidths specifically the reduction in excess theta and the enhancement of alpha-band regulatory dynamics [11]. For attention-related EEG markers, direct comparative evidence demonstrates that both conditions exhibit distinct but overlapping profiles of reduced alpha modulation under cognitive load [10,11]. However, because the therapeutic convergence of these modalities is largely inferred from indirect comparisons across separate ASD-only and ADHD-only trials, it must be interpreted cautiously. Nevertheless, this overlapping mechanism of action hypothetically suggests that for the substantial comorbid ASD + ADHD population, a unified, biomarker-guided approach may be more effective than traditional diagnosis-specific treatments [45,49].

The neurophysiological complexity of comorbid ASD + ADHD presentations further reinforces the need for this unified approach. Direct comparative trials demonstrate that in individuals carrying both diagnoses, EEG profiles can reflect a superposition of disorder-associated signatures—such as elevated frontal TBR often seen in ADHD alongside broadband spectral dysregulation and connectivity disruption typical of ASD [11]. Emerging ML-based frameworks have highlighted that comorbid presentations are particularly poorly served by single-disorder classification models, underscoring the clinical necessity of transdiagnostic biomarker profiling [4]. Furthermore, neurophysiological evidence suggests that altered aperiodic slopes compatible with E/I imbalance may represent a shared mechanistic substrate across both conditions and their comorbid overlap [50], making it a compelling target for unified intervention strategies rather than diagnosis-specific protocols.

Advancing an EEG-guided intervention framework requires explicitly acknowledging the evidential hierarchy on which it rests. We distinguish seven distinct categories of biomarker evidence:

(1) Group-level association: ASD and ADHD populations differ significantly from controls on EEG measures (theta, TBR, connectivity) at the group mean level.
(2) Diagnostic discrimination: ML classifiers achieve high accuracy (~80–95%) distinguishing ASD from ADHD from typical development in research samples.
(3) Test–retest reliability: Candidate EEG markers remain stable within individuals across time a prerequisite for individual-level prediction.
(4) Prediction of clinical outcome: Baseline EEG features predict later symptom severity or functional impairment at the individual level.
(5) Prediction of treatment response: Baseline EEG profile predicts which individual will respond to intervention (vs. non-responder).
(6) Treatment-related change: Intervention produces measurable EEG modulation in responder subgroups.
(7) Mediation of clinical improvement: The EEG change is causally linked to behavioral/symptom improvement (demonstrated via mediation analysis or mechanistic validation).

Current ASD and ADHD literature provides substantial evidence for levels 1–2 (group-level associations and diagnostic classification accuracy). Evidence for level 3 (test–retest reliability) is sparse and inconsistently reported. Evidence for levels 4–7 (individual-level outcome prediction, treatment-response prediction, mediation of clinical improvement) is minimal to absent. The proposed EEG-guided intervention framework in this section therefore rests primarily on levels 1–2 evidence and theoretical mechanistic reasoning, not on demonstrated individual-level predictive validity (levels 4–7). This represents a substantial inferential gap that should be understood as a central limitation of the current state of the field, not a secondary caveat.

Consequently, the provisional scheme below represents an exploratory, hypothesis-generating approach. Pending validation in co-enrolled, active-comparator trials, this scheme offers a theoretical roadmap for prospective testing rather than immediate clinical guidance:

- Mindfulness-First: May be hypothesized for individuals presenting with diffuse “bottom-up” dysregulation, such as high sensory-arousal noise or emotional lability, where global alpha-stabilization is the primary need [51].
- Neurofeedback-First: May be theoretically preferable for those with focal “top-down” executive deficits, particularly when a clearly defined and elevated frontal TBR is present [30,48].
- The Hybrid Model: For complex presentations, a staged approach beginning with mindfulness to cultivate metacognitive awareness, followed by targeted neurofeedback, represents a plausible, yet unverified, strategy for supporting engagement and durable neurophysiological change, although staged protocols of this kind have not yet been directly evaluated [45,49].

## 5. Translational and Technological Extensions

The transition of EEG-informed interventions from laboratory settings to clinical practice necessitates the integration of scalable delivery models and advanced computational frameworks. By leveraging adaptive modalities and machine learning architectures, neurodevelopmental care can move toward a personalized paradigm that accounts for the high degree of heterogeneity within ASD and ADHD populations.

### 5.1. Delivery Modalities and Scalability

The clinical efficacy of mindfulness and neurofeedback interventions in neurodevelopmental disorders may depend on delivery adaptation. Programs should be tailored to account for the unique sensory demands, attention spans, and cognitive profiles of individuals with ASD and ADHD. Effective adaptations for children often integrate multi-modal elements such as yoga-based movement, structured breathing exercises (pranayama), and vocalizations (mantras) to assist in arousal regulation and support regulation of respiratory patterns [52].

Temporal flexibility is a core component of scalability. Research suggests that interventions can range from intensive, long-term programs such as 40-session neurofeedback protocols or 8-week Child-Centered Play Therapy (CCPT) to brief, “just-in-time” 2 min guided breathing exercises delivered via audio recordings [24,39,40]. Furthermore, standard protocols like Mindfulness-Based Stress Reduction (MBSR) have been successfully modified for adults with ASD by shortening weekly sessions and reducing the duration of retreats to prevent sensory or cognitive overload [37]. Programs like MYmind (a concurrent parent–child model) and MindfulTEA (for individuals with intellectual disabilities) further illustrate the trend toward socially embedded and developmentally tiered delivery [52]. These diverse formats aim to ensure that neurophysiological training remains accessible across a wide spectrum of age and ability levels.

The scalability of these interventions ultimately depends not on simplifying their neurophysiological targets but on diversifying the contexts in which those targets are pursued. From intensive, clinician-supervised neurofeedback protocols to brief, audio-guided mindfulness exercises deliverable in home environments, the evidence supports a tiered delivery model in which clinical intensity is calibrated to the individual’s cognitive capacity, sensory tolerance, and ecological circumstances. This flexibility is not merely a logistical convenience it is a prerequisite for ensuring that biomarker-guided intervention remains a realistic option across the full spectrum of neurodevelopmental need, particularly given the frontal lobe maturational dysregulation that characterizes ADHD and influences long-term engagement with training protocols [25].

### 5.2. Machine Learning and EEG-Based Personalization

#### 5.2.1. ML Applications in ASD: From Early Screening to Explainable Biomarker Profiling

The trends in EEG–machine learning studies suggest a shift from traditional disorder classification to interpretable, biomarker-focused personalization. In ASD, systematic reviews have demonstrated that EEG and hybrid EEG-fNIRS workflows can aid early detection through abnormalities in neural oscillations, connectivity, and brain development, however disparity in data preprocessing, electrode montages and model evaluation limit the clinical translation of such evidence [1,2]. Time-frequency and deep learning techniques have demonstrated improved group-level classification accuracy through spectral and time-frequency feature extraction. For example, CNN-based ASD-Net models achieve high accuracy (~85–95%) in distinguishing ASD from typically developing controls on held-out test samples [53]. However, classification accuracy at the group level does not establish clinical predictive validity or utility for individual-level clinical decision-making; prospective validation in independently collected samples with blinded diagnostic confirmation remains limited.

Similarly, machine-learning approaches to early risk stratification using infant EEG features (SVM, Random Forest, SHAP-based) have shown retrospective associations with later diagnosis, suggesting that early neural markers may theoretically support risk identification [54]. However, these analyses remain largely retrospective in design, and prospective validation of whether baseline EEG-based risk predictions improve early intervention timing or outcomes has not been established. Clinical implementation would require prospective validation, standardized preprocessing, and demonstration of utility beyond existing behavioral screening instruments.

Aside from early screening, models dedicated to ASD further expand the focus on explainability and multi-domain feature fusion. Explainable microstate models have demonstrated that temporal instability, spectral distortion and transition unpredictability may represent potential neurophysiological features associated with ASD, supporting the shift from spectral analysis to brain-state mapping [3]. Multidomain fusion models leveraging LSTM-autoencoders, XGBoost and ensemble learning have also shown that time- and frequency-domain features can better capture diverse ASD-related EEG patterns than single-domain features [55]. Low-dimensional and band-specific models based on SHAP, common spatial patterns, quantum support vector machines and optimal alpha/beta band selection indicate that ASD classification can be achieved using minimal biomarker sets rather than dense EEG [56,57]. This is important for clinical applications because low-channel models may enhance the feasibility of wearable or home-based EEG monitoring, and preprocessing-centered studies demonstrate that methods such as ICA, DWT and Butterworth filtering are critical for signal quality, spectral entropy, PSD, and Hjorth-based features used for subsequent ML classification [58]. Recent spatial-structure fusion models also demonstrate the importance of considering EEG as a network rather than independent channels and incorporating local and long-range interactions into ASD classification and future treatment strategies [59].

#### 5.2.2. ML Applications in ADHD: Classification, Connectivity and Treatment Response

In ADHD, studies also favour ML as an adjunct to clinical diagnosis. A review of AI applications in ADHD precision medicine highlights that although EEG-based classifiers typically perform well experimentally, their clinical translation requires independent validation, interpretability, multimodal integration, and care in dealing with small or imbalanced datasets [4]. Deep learning models based on PSD-based brain maps have highlighted frontal and occipital theta-band activity as key discriminative features for ADHD diagnosis, consistent with the well-known role of theta dysregulation in cognitive control [60]. Other raw-EEG based multi-head CNN and recurrent models have also been proposed to enhance cross-subject generalization, which is crucial for such systems to be used by clinicians for unobserved patients, rather than just perform well on a given dataset [61]. Meanwhile, explainable decision-support systems based on Granger-causality inflow and outflow features offer clinically interpretable markers of electrode- and lobe-level dysfunction, enabling ADHD prediction to be associated with intuitive neural communication patterns [62].

ADHD research also shows that multidomain learning can enhance the neurophysiological understanding of a system. GCN approaches using time- and frequency-domain, PLI, coherence, and topological connectivity features indicate that ADHD classification is enhanced by the inclusion of both spectral and network information, rather than simply theta or beta power [63]. Multidomain ensemble-based ML approaches using PSD, fuzzy entropy, mutual-information connectivity, and SHAP feature selection, reveal that increased theta, altered alpha/beta activity, elevated TBR, and reorganized whole-brain connectivity are relevant ADHD markers, confirming a more comprehensive biomarker profile for arousal and attention regulation [64]. Transformers and XGBoost models also demonstrate that attention mechanisms can identify long-term dependencies in EEG data, which may help to adaptively model attention states for closed-loop interventions [65]. Crucially, explainable ML models predicting response to neurofeedback treatment in ADHD bridge the gap between computational modeling and personalization by suggesting that baseline clinical, Behavioral and neurophysiological measures may be used to identify potential treatment responders and non-responders prior to treatment [66].

#### 5.2.3. Toward Personalized Neurodevelopmental Care: A Transdiagnostic ML Framework

**Figure 1:**
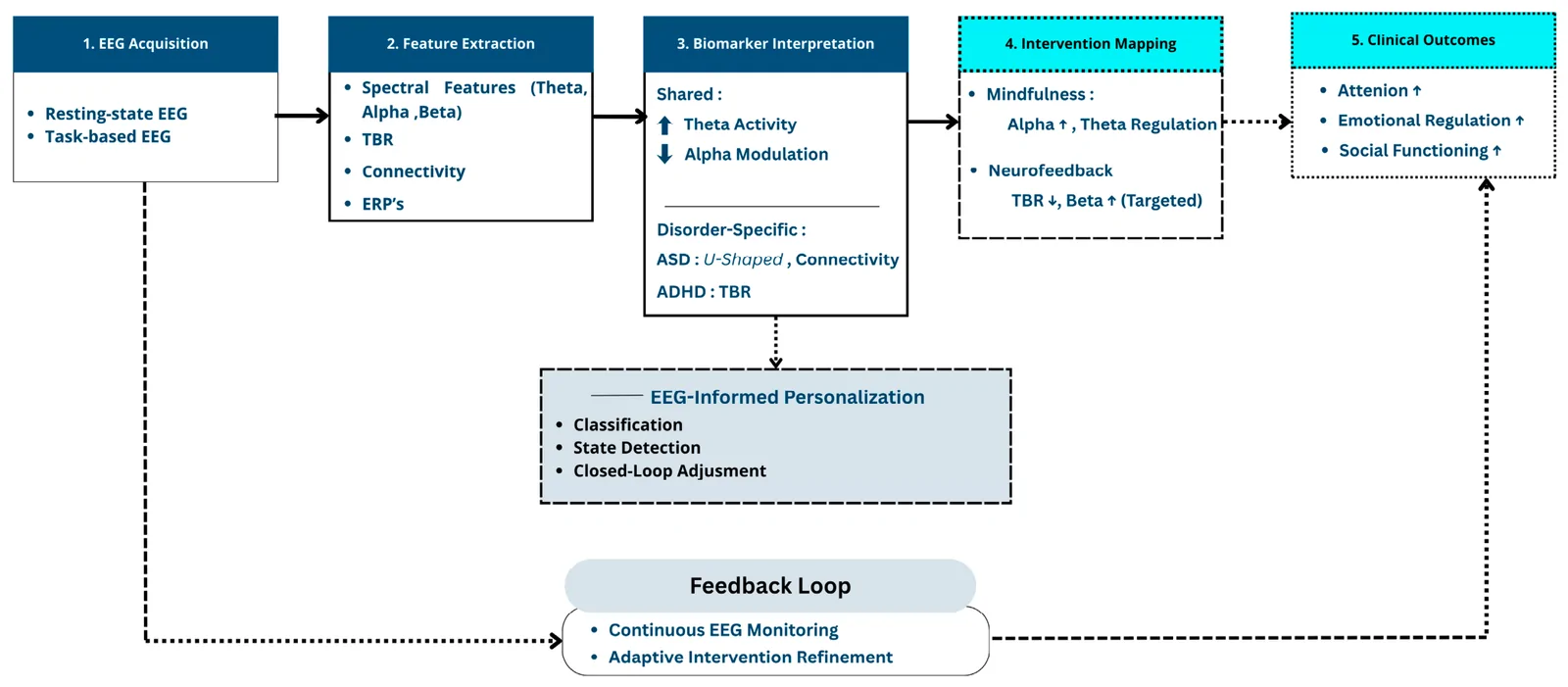
Proposed EEG-Guided Transdiagnostic Intervention Framework for ASD and ADHD. Visual coding: Dark blue/solid boxes and arrows indicate empirically established EEG features and evidence (Steps 1–3). Light cyan/dashed boxes and arrows indicate emerging but unvalidated intervention pathways and clinical outcomes (Steps 4–5). Light gray/dashed elements indicate hypothetical future directions (EEG-Informed Personalization and Feedback Loop). NOTE: This framework is presented as a testable conceptual model based on group-level EEG differences and theoretical mechanistic reasoning. It does not represent validated individual-level clinical decision-making. Prospective validation is required before clinical application, specifically encompassing the following required steps: establishing baseline EEG reliability, defining objective predefined thresholds, prospective randomized assignment, blinded outcome evaluation, direct comparison with non-biomarker-guided clinical allocation, and replication in an independent sample.

The evidence from EEG–ML models suggest that personalization in ASD and ADHD is not about finding a single biomarker, but about the integration of interpretable feature families, such as power, theta/beta ratio, alpha-band activity, connectivity, EEG microstates, entropy, causal-flow features, and temporal dependencies. For ASD, personalization is underpinned by models that reflect growth variability, microstate instability, altered connectivity, and frequency-specific markers. For ADHD, personalization is more closely associated with theta-driven activity, TBR-related features, connectivity reorganization, and prediction of treatment response. Current ML applications in ASD and ADHD primarily operate at evidence levels 1–2: they achieve high classification accuracy for distinguishing diagnostic groups and can identify which EEG features contribute most to group-level discrimination. However, they do not yet demonstrate individual-level predictive validity for clinical outcomes or treatment response (levels 4–5). Translating ML from diagnostic classification to clinical decision-making requires prospective validation showing that EEG-based prediction of individual treatment response outperforms clinically guided assignment. Until such validation is conducted, ML should be understood as a tool for characterizing group-level patterns and identifying potential biomarker targets, not yet as a validated biomarker translation tool for individual clinical decisions. Figure 1 illustrates this proposed EEG-guided transdiagnostic intervention framework, depicting the progression from EEG acquisition and feature extraction through biomarker interpretation to intervention mapping and clinical outcomes, with integrated machine-learning-based personalization and feedback mechanisms.

As summarized in Table 3, work in EEG-ML has emerged to support a move from single model diagnosis to clinically relevant personalization. In ASD, personalization is enhanced by models that represent the early developmental risk, microstate instability, time-frequency pathology, and altered connectivity. In ADHD, the most relevant models highlight theta predominance, TBR markers, connectivity repatterning and neurofeedback responsiveness. Thus, the value of ML is not only in the improvement of diagnostic accuracy but in translating an individual’s EEG profile into decision-making that is relevant for intervention, such as predicting whether he or she is more likely to benefit from mindfulness-based regulation, specific neurofeedback, or a combination of self-regulation approaches.

## 6. Current Challenges and Research Gaps

The primary barrier to clinical translation is methodological heterogeneity. Variability in recording parameters, electrode montages, and analytical pipelines makes cross-study comparisons difficult and risks misinterpreting technical inconsistency as neurophysiological divergence. Furthermore, the lack of standardization in intervention design particularly the scarcity of EEG-informed mindfulness trials in ASD limits our ability to verify whether clinical gains are supported by robust neural shifts.

Crucially, the current literature suffers from a “representation gap”. Available evidence is sparse for comorbid ASD + ADHD populations, adults, and females, which weakens the ecological validity of existing transdiagnostic frameworks and limits their application in real-world, diverse clinical settings.

The absence of conventional preprocessing standards particularly regarding artifact rejection, epoch length, and frequency band definitions further limits cross-study comparability even within the same diagnostic category.

## 7. Limitations of the Present Review

This review has several substantive limitations that constrain its clinical applicability. It was conducted as a structured integrative synthesis rather than a systematic meta-analysis, and screening was not performed in duplicate. Inferential Gap Between Evidence Levels.** Most evidence reviewed in this manuscript operates at evidence levels 1–2 (group-level associations and diagnostic discrimination via ML classification), whereas the proposed intervention framework claims to operate at levels 4–5 (individual-level treatment-response prediction and personalized assignment). No studies in the current ASD or ADHD literature have demonstrated that EEG-guided individual assignment to interventions produces superior clinical outcomes compared with clinically guided or randomly assigned control conditions. Evidence for level 3 (test–retest reliability of individual EEG markers across time) is sparse and inconsistently reported, and evidence for level 7 (mechanistic mediation linking EEG change to clinical improvement) is absent. Consequently, the proposed EEG- guided intervention framework rests on an unvalidated inferential leap from group-level patterns to individual-level predictions. A major substantive limitation of the current field is the heavy reliance on indirect cross-diagnostic inferences: true direct comparative studies (such as [10,11]) constitute only ~3% of the synthesized evidence base, and empirical investigations of comorbid ASD + ADHD cohorts remain virtually absent outside of isolated studies (e.g., [11]). Heterogeneity in EEG acquisition, preprocessing pipelines, and frequency-band definitions across single-diagnosis studies precluded quantitative pooling. Furthermore, most biomarker findings derive from group-level associations, which do not guarantee individual-level therapeutic prediction. A group-level EEG difference does not establish a causal mechanism, nor does it ensure that neuromodulation of that specific feature will yield clinical improvement for the individual. Accordingly, the intervention-selection framework proposed here should be regarded strictly as hypothesis-generating rather than as clinical guidance.

Finally, significant methodological heterogeneity across the cited literature including variations in frequency-band definitions, absolute versus relative power metrics, and resting-state recording conditions (eyes open versus eyes closed) was retained according to the original publications, representing an ongoing confound in synthesizing group-level spectral profiles.

## 8. Future Directions

Future research must prioritize large-scale, comparative randomized controlled trials that evaluate mindfulness, neurofeedback, and hybrid models in tandem. Such trials are essential to determine which modality is superior for specific EEG profiles. Additionally, shifting toward longitudinal designs will help clarify whether observed EEG normalization reflects temporary state shifts or stable neuroplastic adaptations. The next translational frontier lies in the integration of passive wearable EEG with machine-learning-driven, “just-in-time” adaptive interventions. By leveraging consumer-grade sensors and closed-loop analytics, care can move beyond the laboratory to provide real-time, personalized support in ecological environments. While MEG findings from other neurological disorders provide methodological examples of how electrophysiological dynamics can predict individual clinical features, EEG is uniquely suited for this repeated, ecological, and intervention-related monitoring. Artificial intelligence will be critical to integrate these multimodal data streams, estimate individualized parameters, and update predictions longitudinally. Validating these systems in real-world neurodevelopmental populations and establishing appropriate regulatory frameworks remains the primary challenge for clinical deployment.

### Individual EEG Fingerprinting and Fast Brain Dynamics

While the present review has prioritized group-level EEG markers, an important emerging direction involves subject-specific EEG fingerprinting and fast brain dynamics. Individual EEG fingerprints reproducible electrophysiological signatures sufficiently distinctive to identify a person from their own prior recordings have demonstrated above-chance identifiability in healthy populations [67,68] and show promise for capturing individual neurobiological variability in ASD and ADHD, where symptom overlap and interindividual heterogeneity limit group-level diagnostic utility. Complementing fingerprinting, dynamic measures of temporal network reconfiguration, brain-state transitions, and functional repertoire flexibility characterize how individuals adaptively reconfigure their neural systems in response to changing demands.

A critical advantage of fingerprinting approaches is their direct utility for the trait/state distinction introduced in this review’s limitations. By repeatedly assessing the same individual under standardized, controlled conditions, fingerprint stability can be quantified, and state-dependent fluctuations (from sleep, hormones, medication, and arousal) can be isolated from stable individual characteristics. Methodological evidence from multiple sclerosis, amyotrophic lateral sclerosis, and mild cognitive impairment supports the clinical relevance of these approaches; however, developmental validation in ASD and ADHD is currently lacking.

Future research should: (1) establish whether individual EEG fingerprints remain stable across development, and whether fingerprint-based classification of ASD versus ADHD remains accurate despite maturational changes in spectral profiles; (2) directly compare fingerprinting and dynamic approaches with conventional group-level markers for predicting symptom progression and intervention response; (3) characterize the trait and state components of individual fingerprints through repeated measurement under controlled conditions; (4) investigate whether fingerprint-guided intervention selection produces superior outcomes compared with group-level or clinically guided approaches; and (5) explore the integration of these dynamic EEG measures with emerging “Virtual Brain” and digital-twin frameworks.

## 9. Conclusions

EEG research in ASD and ADHD reveals apparent convergence patterns characterized by low-frequency theta excess and impaired alpha-band modulation in many though not all individuals. While disorder-specific signatures like the ASD “U-shaped” profile (heterogeneous across development) and ADHD frontal TBR persist, shared deficits in attention and self-regulation support the investigation of biomarker-guided interventions. However, substantial individual heterogeneity within both conditions limits the applicability of group-level signatures to individual-level clinical decision-making absent baseline confirmation.

This review establishes that EEG research has identified candidate biomarkers and group-level neurophysiological patterns relevant to ASD and ADHD (evidence levels 1–2). Both mindfulness and neurofeedback produce measurable EEG modulation in responder subgroups (evidence level 6), and conceptually mechanistic pathways exist linking these EEG changes to symptom targets. However, the evidence required to support individual-level, biomarker-guided treatment assignment (evidence levels 4–5) remains absent. Moving from diagnostic categories toward EEG-informed care will require prospective validation demonstrating that: (1) individual EEG markers have test–retest reliability across clinically relevant timeframes (level 3); (2) baseline EEG profiles predict individual treatment response better than clinical judgment (level 5); and (3) EEG-guided assignment produces superior clinical outcomes compared with clinically guided or sham-controlled assignment (level 7). Until such validation occurs, the proposed biomarker-guided framework should be understood as a hypothesis-generating model for future testing, not as ready for clinical implementation.

## Figures and Tables

**Table 1:** Scope of recent EEG-based computational reviews in neurodevelopmental disorders and the complementary focus of the present synthesis.

Ref.	Domain	Primary Focus of Prior Work	Complementary Focus Here
[1]	Scope	Establishes ASD-versus-typical classification performance using deep learning	Extends comparison to ASD-versus-ADHD spectral profiles
[4]	Comorbidity	Prioritises diagnostically homogeneous cohorts to control dataset variance	Foregrounds comorbid ASD + ADHD neurophysiological signatures
[5]	Intervention	Advances classification metrics (AUC, F1) for explainable EEG models	Maps identified signatures onto mindfulness and neurofeedback targets
[6]	Feature selection	Develops evolutionary and massive feature-extraction methods for biomarker discovery	Applies interpretable feature families to adaptive clinical state detection
[7]	Personalization	Spans psychiatric diagnoses broadly using resting-state EEG	Focuses on neurodevelopmental intervention selection
[8]	Clinical scope	Provides umbrella-level overview of AI support for childhood diagnosis	Provides band-level spectral synthesis (alpha, theta, TBR) for treatment guidance
[9]	Connectivity	Identifies shared functional connectivity signatures across neurodevelopmental disorders	Links shared connectivity profiles to unified intervention strategies

**Table 2:** Summary of EEG Features Reported in ASD and ADHD Studies.

Feature	ASD	ADHD	Shared/Overlapping	Evidence Quality
Delta/Theta power	Increased (especially early development, “U-shaped” profile)	Increased (frontal theta hallmark)	Shared low-frequency excess	Robust (ASD), Moderate (ADHD)
Alpha power	Decreased (posterior, correlates with social deficits)	Decreased or normal; reduced suppression on task	Reduced alpha modulation under load	Moderate (both)
Beta power	Increased or normal (excess beta in some)	Decreased (especially inattentive subtype)	Divergent: Excess (ASD) vs. Deficit (ADHD)	Limited/Inconsistent
Gamma power	Increased or inconsistent	Rarely reported	Under-investigated in both	Very Limited
Theta/Beta Ratio (TBR)	Variable, emerging utility	Often increased (widely studied biomarker)	Elevated in comorbid presentations	Robust (ADHD), Limited (ASD)
Epileptiform activity	Reported in a subset of cases (20–85% across studies, reflecting differences in recording state and number of recordings)	Rarely reported	Disorder-specific (ASD hallmark)	Moderate (heavily influenced by methodology)
Aperiodic (1/f) Slope	Flatter slope (E/I imbalance)	Flatter slope (E/I imbalance)	Shared E/I dysregulation	Limited (emerging evidence)

**Table 3:** Proposed EEG–ML Personalization Framework: Biomarker Inputs, Model Strategies, and Clinical Applications in ASD and ADHD. NOTE: This framework operates primarily at evidence levels 1–2 (group-level associations and diagnostic discrimination). Individual-level treatment-response prediction (evidence level 5) remains unvalidated. This table represents a hypothesis- generating model requiring prospective validation in active-comparator randomized controlled trials before clinical implementation.

Personalization Concept	EEG Biomarkers/Inputs	ML/DL Models	ASD/ADHD Relevance	Clinical Personalization
Early-risk screening	Infant EEG power, nonlinear EEG features, EEG/fNIRS developmental biomarkers	SVM, RF, DL classifiers, systematic ML/DL frameworks	Mainly ASD; emerging ADHD precision-screening relevance	Supports earlier identification of neurodevelopmental risk before stable Behavioral diagnosis, allowing earlier monitoring and intervention planning [1,2,54].
Spectral biomarker profiling	Theta, alpha, beta, gamma power, TBR, PSD brain maps, optimal alpha/beta bands	SVM, Siamese CNN, SHAP-guided feature selection, Grad-CAM	ASD: alpha/beta/gamma abnormalities; ADHD: theta/TBR dominance	Helps identify whether the individual profile is dominated by sensory-arousal dysregulation, alpha/beta imbalance, or frontal theta excess, guiding mindfulness or neurofeedback targets [56,57,60,64].
Time-frequency representation learning	Spectrograms, scalograms, SPWVD-TFD images, dynamic oscillatory patterns	CNN, DenseNet, ResNet, ASD-Net, transformer-based models	Strongly useful for ASD; also relevant for ADHD temporal attention modeling	Converts raw EEG into visual or sequence-based representations that capture both transient and frequency-specific abnormalities for individualized monitoring [53,65].
Connectivity and network modeling	Functional connectivity, coherence, PLI, Granger causality, inflow/outflow, electrode-level network patterns	GCN, XGBoost, logistic regression, SHAP, LIME, PDP	ASD: disrupted long-range integration; ADHD: altered attention-network connectivity	Enables model outputs to be interpreted as affected regions, lobes, or network pathways, supporting targeted neurofeedback or coherence-training protocols [59,62,63,64].
Dynamic brain-state interpretation	EEG microstates, temporal instability, transition unpredictability, complexity features	XGBoost, SHAP, multidomain microstate analysis	Mainly ASD; useful for future transdiagnostic monitoring	Moves personalization from static diagnosis to brain-state tracking, allowing clinicians to monitor regulatory stability and intervention-related neural change [3].
Multidomain fusion and decision support	Combined time-domain, frequency-domain, entropy, connectivity, spatial, behavioral, and clinical features	LSTM-autoencoder, ensemble fusion, multi-head CNN-RNN, XGBoost, RF	ASD and ADHD	Integrates multiple biomarker families into a single decision-support layer, improving robustness for heterogeneous and comorbid presentations [4,55,58,61].
Treatment-response prediction	Baseline clinical, behavioral, personality, demographic, and neurophysiological predictors	RF, SVM, LR, ANN, AdaBoost, SHAP	Most developed in ADHD neurofeedback studies	Directly supports personalized intervention selection by identifying likely responders and non-responders before neurofeedback or hybrid intervention begins [66].

## Data Availability

No new data were created or analyzed in this study. Data sharing is not applicable to this article.
